# Bone Mineral Density and Osteoporosis after Preterm Birth: The Role of Early Life Factors and Nutrition

**DOI:** 10.1155/2013/902513

**Published:** 2013-04-10

**Authors:** Claire L. Wood, Alexander M. Wood, Caroline Harker, Nicholas D. Embleton

**Affiliations:** ^1^Child Health, Newcastle Hospitals NHS Foundation Trust, Newcastle upon Tyne NE1 4LP, UK; ^2^Orthopaedic Department, Wansbeck General Hospital, Woodhorn Lane, Ashington, Northumberland NE63 9JJ, UK; ^3^Newcastle University, Framlington Place, Newcastle Upon Tyne NE2 4HH, UK; ^4^Newcastle Neonatal Service, Newcastle Hospitals NHS Foundation Trust, Newcastle upon Tyne NE1 4LP, UK; ^5^Institute of Health and Society, Newcastle University, Framlington Place, Newcastle Upon Tyne NE2 4HH, UK

## Abstract

The effects of preterm birth and perinatal events on bone health in later life remain largely unknown. Bone mineral density (BMD) and osteoporosis risk may be programmed by early life factors. We summarise the existing literature relating to the effects of prematurity on adult BMD and the Developmental Origins of Health and Disease hypothesis and programming of bone growth. Metabolic bone disease of prematurity and the influence of epigenetics on bone metabolism are discussed and current evidence regarding the effects of breastfeeding and aluminium exposure on bone metabolism is summarised. This review highlights the need for further research into modifiable early life factors and their effect on long-term bone health after preterm birth.

## 1. Introduction

Preterm birth accounts for 5–10% of births in the UK. Worldwide almost 10% of babies are born preterm, representing more than 15 million births every year [1]. Preterm birth is defined by the World Health Organisation (WHO) as all live births before 37 completed weeks gestation. Preterm can be further subdivided into extremely preterm (<28 weeks), very preterm (28–32 weeks), and moderate preterm (32–<37 completed weeks) [1]. A preterm baby faces many challenges. Feeding problems are almost inevitable in the very preterm group as a coordinated suck and swallow is not established until around 34 weeks corrected gestation. Extremely preterm infants and those who are unwell may require IV fluids or a period of total parenteral nutrition before full feeds can be established. Many preterms born at less than 32 weeks will have some degree of respiratory distress syndrome (RDS), due to lung immaturity, and may require ventilatory support. Giving antenatal steroids reduces the incidence and severity of respiratory and other complications. The use of supplemental oxygen increases the risk of retinopathy of prematurity and may exacerbate oxidant damage in many organs and tissues but is vital for improved survival.

Despite these challenges, survival has improved dramatically in the last few years, especially in developed countries. More than 50% of babies born at 24 weeks gestation regularly survive long term with improved nutrition being one potential factor contributing to these improvements. As this cohort of survivors reaches middle age the impact of preterm birth on long-term metabolic outcomes such as bone mineral density will become increasingly important.

Osteoporosis is characterised by the depletion of bone mineral mass, combined with bone microarchitecture deterioration and a resultant increased fracture risk [2]. It is one of the most prevalent skeletal disorders; with estimates that up to 30% of women and 12% of men over the age of 50 are affected [3], it has a similar lifetime risk to coronary heart disease [4]. Bone mineral density in adulthood depends predominantly on growth and mineralisation of the skeleton and the resultant peak bone mass achieved and then, to a lesser extent, on the subsequent loss. Longitudinal studies of girls suggests that this peak is reached about 30 years of age [5]. For each standard deviation decrease in bone mineral density, fracture risk doubles in girls, similar to the risk in postmenopausal women [6].

It is estimated that osteoporosis affects 3 million people in the UK and results in 250,000 fractures annually [2]. It has vast public health consequences due to the morbidity and mortality of the resulting fractures and the associated healthcare expenditure. As there is no cure, it is important to identify early life influences on later bone mineral density, which may aid the development of interventions to optimise bone health and reduce osteoporosis risk.

We present a review of the current literature regarding early life factors and the impact of nutrition on bone mineral density and bone health after preterm birth, in order to inform further research and highlight current challenges facing the clinicians responsible for this cohort. 

## 2. Bone Mineral Density (BMD) Programming in Term Infants

There is strong evidence linking early life exposures and later peak bone mass in childhood, for example, the contributions of physical exercise both in utero and childhood, cigarette smoking during pregnancy, and diet and endocrine status in childhood [7, 8]. Bone mineral density shows strong tracking during childhood and adolescence growth and into adulthood. A reduced peak BMD in childhood is associated with increased fracture risk and has been proposed as one of the best predictors of later life fracture risk in females [9]. Gender also influences neonatal bone composition at term with males attaining greater bone area, bone mineral content (BMC), and BMD than females after adjustment for gestation [10]. In addition to factors influencing peak bone mass during childhood and adolescence, evidence is growing that bone mineral density and thus osteoporosis risk can be modulated during intrauterine and infant life [11]. A retrospective study involving term infants demonstrated independent effects of birth weight and weight at one year on bone size and strength during the sixth and seventh decades after adjustment for confounding lifestyle factors [12]. These associations may reflect the intrauterine programming of skeletal development [13] and its subsequent tracking throughout the lifecourse. 

Research also suggests that some of the predisposition for osteoporosis can be attributed to polygenic genetic inheritance. For example, polymorphisms in vitamin D and oestrogen receptor genes and collagen coding genes have been implicated [14]. It is likely that the genes that determine an increased risk of osteoporosis will vary among people of different ethnic backgrounds. In the future, genomic studies may provide information regarding the susceptibility of osteoporosis and likely treatment response and may become an adjunct to clinical management.

## 3. The Developmental Origins of Health and Disease (DOHaD) Hypothesis and Programming of Bone Growth

The Developmental Origins of Health and Disease (DOHaD) hypothesis suggest that nutritional imbalance during critical windows in early life can permanently influence or “programme” long-term development and disease in later life [15]. Much of the original work was by Barker who reported the relationship with low birthweight, used as a proxy for fetal growth, with coronary heart disease [16, 17]. It became apparent, however, that these mechanisms and effects were not restricted to fetal life and that nutrition and growth in infancy (and perhaps in later childhood) were also crucial, leading to the incorporation of elements of evolutionary biology and the adoption of the term DOHaD. 

Recently, research has linked birth weight, birth length, and placental weight to later osteoporosis risk [18–20]. Known predictors of osteoporosis risk comprise genetic predisposition and environmental influences such as diet and exercise. However, a significant portion of BMD variance remains unexplained [19]. It is proposed that this remaining variation results from the programming of systems controlling skeletal growth trajectory during critical growth periods [13]. 

## 4. Epigenetics and Bone Metabolism

Many of the programming effects may be modulated by epigenetic mechanisms. Epigenetics is the study of mitotically heritable alterations in gene expression potential that are not caused by changes in DNA sequence. The classic examples are DNA methylation and histone acetylation [21]. These processes do not alter the nucleotide sequence in DNA but result in differences in gene expression and transcription and may also involve post-transcriptional effects on other processes such as protein translation. Early life growth and nutritional exposures appear to affect the “cellular memory” and result in variation in later life phenotypes. Much of this work is still in the early stages but initial data suggest that epigenetic mechanisms may underlie the process of developmental plasticity and its effect on the risk of osteoporosis. One of the models that have been postulated is the role of maternal vitamin D status and postnatal calcium transfer. Calcium and vitamin D are vital nutrients in bone development. Early work concerning methylation and vitamin D receptors and placental calcium transporters suggests that epigenetic regulation might explain how maternal vitamin D levels affect bone mineralisation in the neonate [21]. Much of the current research is in animal models, but if the changes can be replicated in humans, epigenetic or other biomarkers may provide risk assessment tools to enable targeted intervention to those at greatest risk of osteoporosis.

## 5. Metabolic Bone Disease of Prematurity

The preterm population is particularly susceptible to metabolic bone disease for two key reasons: firstly, 80% of fetal bone mineral accumulation occurs during the last trimester of pregnancy, with a surge in placental transfer of calcium, magnesium, and phosphorus to the neonate [22]. A preterm infant who spends this period without the placenta and the associated regulatory maternal environment will therefore have lower BMD and significantly lower bone mineral content than an infant born at term. 

Secondly, providing adequate nutrition to preterm infants can be extremely challenging. Most extremely preterm infants (<32 weeks gestation) require support with parenteral nutrition (PN) because of complex factors including metabolic “immaturity” (that may limit nutrient intake) and a delay in establishing enteral feeds. In addition, solubility issues with PN solutions mean it is impossible to provide sufficient mineral via the parenteral route alone. Maternal breast milk is associated with a range of benefits in the short-term (e.g., reduction in the incidence of necrotising enterocolitis, a potentially fatal illness associated with milk feeds) and long-term (e.g., improved cognitive outcome) but alone will not meet nutrient requirements without fortification. Therefore as well as being born with a mineral deficit, the often stormy neonatal course and nutritional practicalities of providing adequate mineral intake means that many preterm infants develop osteopenia of prematurity.

As preterm infants grow, mineral uptake is compromised through the low content in unfortified breast milk (especially phosphate) and inefficient absorption due to an under-developed gastrointestinal tract [9]. This results in a greater loss of long bone density than observed in term infants and further increases the risk of metabolic bone disease [9]. Ex utero living conditions also mean it is more difficult for infants to move and stress their bones as they would have done in-utero [23, 24]. As well as mineral compromise, lower BMD in preterms is also a consequence of other factors such as steroid use [25], respiratory compromise [25], and infection [18] which may damage bone trabeculae. Although metabolic bone disease of prematurity is often asymptomatic and self-limiting [9], concern remains that under-mineralisation during such a critical period could increase the risk of childhood fracture and cause reduced peak bone mass [26] and therefore an increased risk of future osteoporosis.

## 6. The Effects of Prematurity on Adult BMD

There is conflicting data regarding the long-term consequences of preterm birth on the skeleton and the potential for peak BMD compared to their term counterparts. Preterm infants are known to have a lower bone mass [27], BMD [26] and BMC [25] at the corrected age of term, as well as a lower weight and ponderal index [26]. A study of 7-year-old boys showed greater measures of cortical thickness, whole body BMC, and hip BMD in term compared to preterm boys after adjustment for weight, height and age. These differences remained after adjustment for birth weight, length of neonatal hospital stay, and current activity level [28]. A study by Fewtrell et al. in 2000 [29] found former preterm infants who were followed up at around 10 years of age were shorter, lighter, and had lower BMC than controls. These differences continue through childhood and possibly persist until puberty [25, 28], although results are difficult to interpret due to the confounding effects of puberty and the interaction with bone size and later BMD. In a study by Backström et al., individuals who were born preterm were assessed with computerized tomography as young adults. Lower bone strength was demonstrated at the distal tibia and radius compared to age and sex matched controls [30]. This effect was more pronounced in males and remained after adjustment for potential confounders.

Several studies have failed to demonstrate an association between preterm birth and later bone strength, although all of these [28, 31, 32] were undertaken in small populations. A possible explanation for the variation in study results may be in the timing of follow-up as catch up in bone mineralisation may occur primarily in late childhood and adolescence. Other studies have found that although preterms were smaller, their BMD was appropriate for size. Adults who were born preterm may be shorter than their term born counterparts. As some studies may not have made appropriate adjustments for current size it is difficult to determine whether BMD is appropriate for current size or not [28].

## 7. Early Nutrition and Growth Influences on Bone Metabolism after Preterm Birth

Several maternal factors are known to have a negative impact on neonatal growth and skeletal mineralisation in term infants. Although not discussed in detail here, examples are shorter maternal height, low parity, smoking during pregnancy, low fat stores [33, 34], and low vitamin D exposure [9, 22].

There is conflicting data regarding the influence of birthweight on later BMD. Low birthweight (LBW) is defined by WHO as <2500 g [1]. LBW is usually a consequence of being preterm or small for gestational age (i.e., born with a birthweight on less than the 10th centile). Some studies suggests that very low birth weight (VLBW, <1500 g birthweight) infants, whether preterm or not, attain a suboptimal peak bone mass in part due to their small size and subnormal skeletal mineralisation [31]. A recent study by Callreus et al. highlighted the long-term influence of birthweight on bone mineral content but found an absence of association of birthweight with bone density once adult body weight was also taken into consideration [39]. The Hertfordshire cohort study involving over 600 subjects showed that birthweight was independently associated with bone density at 60–75 years of age. Although another study found no association with preterm birth and peak bone mass [35], an effect of being small for gestational age was apparent, suggesting a proportion of later bone mass is determined by fetal growth. Further research has also shown a significant association between shorter gestation and adverse skeletal outcomes [31]. 

Several studies in infants have shown the influence of early growth on later bone health in those born preterm. In a study by Cooper et al., those who were lightest at 1 year of age had the lowest BMC [22]. In a further study, weight gain during the first two years of life predicted BMD at age 9–14 [40]. Fewtrell et al. also showed a positive association of body weight and height at both premature birth and 18 months with bone size, BMC, and BMD at aged 8–12 years [36]. It was hypothesised that those with the most substantial increase in height between birth and follow-up showed the greatest bone mass. They also demonstrated that birth length alone was a strong predictor of later bone mass, and it was suggested that optimising linear growth early may be beneficial to later bone health. Although conducted with a large cohort (*n* = 201), few measurements were taken after discharge and dual-energy X-ray absorptiometry (DXA) analysis was only taken at 8–12 years. As a result, changes in growth and corresponding bone mass at potentially critical epochs of infancy were not measured. 

Optimising early growth through nutritional interventions generates positive and lasting effects on bone mineralisation [28] and it is hypothesised that this may partially counteract preterm bone deficits. A systematic review by Kuschel and Harding in 2009 showed that fortifying the nutrition of preterm babies improves growth and bone mineral aggregation [41].

Lieben et al. [42] and Kanazawa et al. [43] discuss an interaction between bone and glucose metabolism involving adipocyte-originated leptin and osteoblast-derived osteocalcin. They postulate that healthy bone matrix protein increases insulin sensitivity in other tissues and that people with metabolic syndrome who are insulin resistant also have poorer bone quality and increased risk of osteoporotic fracture. The “metabolic syndrome” involves many biological systems, but insulin sensitivity or resistance is perhaps the area subject to the most detail study in later life. This interaction is potentially a very important one; those who were born preterm appear to be at a higher risk for metabolic syndrome in later life and studies examining the influence of birth weight on later health consistently show that in low birth weight born adults, there is decreased insulin sensitivity. The critical period in the preterm determining later insulin resistance is unclear at present. Bazaes et al. found that low preterm birthweight was associated with impaired insulin sensitivity [44], which supports Barker's hypothesis. Singhal et al. [45] showed that preterm infants who received higher nutrient intakes during the first 2 weeks of life had higher levels of insulin resistance in adolescence. These studies may be showing a potential adipocyte-osteocalcin interaction and suggest that the relationship between nutrition and later bone and metabolic health is complex, and this is an area that clearly warrants further research. 

## 8. Aluminium and Bone Mineral Density

Aluminium has no active role in the human body but is inadvertently ingested in the preterm for several reasons. Firstly preterms are exposed to high levels of aluminium in standard parenteral nutrition (PN) regimes. The current trend is for early PN to optimise early growth and associated neurocognitive function. Most aluminium is accumulated through unavoidable contamination via calcium gluconate stored in glass vials. In adults, this aluminium load is probably adequately dealt with by the kidneys, but the premature infant's renal system is relatively immature so accumulation occurs. Adverse effects of aluminium in bone have been seen in uraemic adults and there are now studies showing that infants who received aluminium-depleted PN had significantly higher BMC of the lumbar spine [46]. A direct effect on bone structure is unlikely as bone will have been remodeled several times by adulthood, but it is thought that the presence of aluminium may modify the response of bone cells to stimuli such as when loading forces are applied through exercise.

## 9. Effects of Breastfeeding on Bone Metabolism

There is conflicting evidence as to whether breastfeeding has a protective role in the primary prevention of osteoporosis. In some studies, such as that of Fewtrell et al., breast milk consumption was found to result in higher adult BMD [37] despite the milk being unfortified and having a lower mineral content than formula. This suggests a possible role for beneficial non-nutrient components such as growth factors. In another study, bone mass at follow-up age of approximately 10 years was positively associated with the duration of breastfeeding [47], yet other studies have shown no benefits at a similar age [48, 49]. Other studies have not demonstrated an ongoing relationship in adulthood between breastfeeding and bone mass [22]. Given the known benefits of breastfeeding and the lack of proven negative association, it seems prudent to strongly encourage breastfeeding, despite slower infant growth trajectories.

## 10. Vitamin D and Bone Mineral Density

It is difficult for the preterm infant to match the in-utero accretion of minerals. Calcium absorption depends on calcium and vitamin D intakes and phosphorus levels, which affect calcium retention. In clinical practice, very few babies need calcium supplementation if they receive either a preterm formula or breastmilk along with breastmilk fortifier [50]. Suboptimal maternal vitamin D levels have been reported from many sources [51]. There are few studies in the preterm population but data from term infants clearly show maternal vitamin D insufficiency to be associated with adverse BMD both in infancy and later follow-up [52]. Considering the prevalence of vitamin D deficiency in pregnant mothers, the European Society of Paediatric Gastroenterology Hepatology and Nutrition (ESPGHAN) committee recommends vitamin D supplementation in the region of 800–1000 IU per day to preterm infants to rapidly correct low fetal plasma levels and that they should be continued through infancy [53]. 

## 11. Limitations of the Current Evidence

Little is known concerning the early life control mechanisms for bone development [26] and the lack of prospective research in this area has been highlighted [30]. The potential for confounding in observational studies is also an important consideration. Poor nutrition is often an inevitable consequence in the sickest neonate who in turn will be more likely to have a poorer metabolic outcome. A 2011 meta-analysis stated that research from a variety of populations may clarify inconsistencies concerning the relationship between early life events and subsequent bone health [19], and there are few studies relating gestational length to adolescent BMD [9]. There is a need for longitudinal studies utilising randomised controlled trials of preterm infants where possible, and providing detailed information on early life exposures as well as bone measurement data.

One of the greatest challenges of longitudinal cohort studies, especially in children, is the attritional losses over time. In addition, much of the current data available is from preterm infants recruited to studies in the 1980s, an era pre-dating the widespread use of antenatal steroids and surfactant therapy; two of the key practices that have had the most dramatic effects on long term survival. As cohorts of preterm born adults reach middle age, their risk of osteoporosis and their antecedent risks factors will become increasingly apparent.  Table 1 summarises some of the key research on BMD and osteoporosis after preterm birth.

## 12. Conclusion

As survival rates continue to improve, the long term effects of premature birth become increasingly important. Only decades of future follow-up will truly ascertain the risk of osteoporosis and fracture after preterm birth. Because there is no cure for osteoporosis, preventative measures are important to minimise risk in this susceptible population. Genetic and intrauterine environmental factors that influence fetal growth trajectory have long-term consequences on body composition. Clearer identification of risk factors and refinement of biomarkers for later bone health will enable earlier preventative strategies. Reduction of the exposure of preterm infants to aluminium is an urgent research and clinical priority. Breastfeeding along with appropriately formulated breastmilk fortifiers to ensure adequate mineral intake and optimal growth should be strongly encouraged. As early mineral deficiency and metabolic bone disease are often asymptomatic during neonatal period, careful follow-up is required to identify at risk groups. Targeted prevention, early diagnosis and appropriate timely treatment may then significantly reduce the individual, health service, and societal burden of osteoporosis in the future.

Key learning points are as follows.

(i) There are conflicting data regarding the effects of preterm birth and/or LBW on later BMD.
(ii) Need for further research into modifiable early life factors and long-term bone health.
(iii) Breastfeeding (with appropriate fortifiers) and vitamin D supplementation may have long term benefits on BMD in preterm infants.
(iv) Reduction of aluminium exposure in preterm infants is an urgent priority.

## Figures and Tables

**Table 1:** Summary of key papers on BMD and osteoporosis after preterm birth.

Author	Year	Cohort type	Study design	Findings
Rigo et al. [9]	2007	Preterm and term	Review	Greater loss of BMD in preterms than in terms during neonatal period. Maternal vitamin D exposure affects bone health in the newborn.
Bowden et al. [25]	1999	Preterm and term	Retrospective cross-sectional	Preterm infants have reduced bone mineral mass in conjunction with reduced growth and hip BMD aged 8 years.
Hovi et al. [31]	2009	LBW infants	Cohort	VLBW young adults have reduced peak BMD than their term peers.
Ahmad et al. [26]	2010	Preterm and term	Prospective	Preterms had lower body weight, length and BMD at term compared to term-born infants.
Abou Samra et al. [28]	2009	Preterm and term	Cross-sectional	Term males have greater bone size and mass than preterm males at follow-up aged 7 years.
Backström et al. [30]	2005	Preterm and term	Cross-sectional	Preterms have smaller cross-sectional bone dimensions in adulthood than terms.
Dalziel et al. [35]	2006	Preterm	RCT with longitudinal follow-up	Antenatal steroids did not affect peak bone mass. LBW and short gestation predicted reduced adult height. Slow fetal growth predicted lower bone mass.
Fewtrell et al. [36]	2000	Preterm	Longitudinal	Bone mass at 8–12 years is related to current size. Linear growth important in maximising bone mass.
Fewtrell et al. [37]	2009	Preterm	Longitudinal	Infant diet does not affect peak bone mass.
Breukhoven et al. [38]	2011	Preterm	Cross-sectional	Preterm birth does not affect BMD in young adults.
